# Postoperative adjuvant immunotherapy for high-risk hepatocellular carcinoma patients

**DOI:** 10.3389/fonc.2023.1289916

**Published:** 2023-12-15

**Authors:** Wei-Qiao Zhang, Qiao Zhang, Li Tan, Zhi-Feng Guan, Feng Tian, Hong-Tao Tang, Kun He, Wei-Qiang Chen

**Affiliations:** ^1^ Guangdong Medical College, Zhanjiang, China; ^2^ Department of Emergency, Zhongshan Hospital, Zhongshan, China; ^3^ Department of General Surgery, Zhongshan Hospital, Zhongshan, China

**Keywords:** HCC, high-risk recurrence factors, anti-PD-1 antibodies, postoperative adjuvant therapy, biomark

## Abstract

**Background and aim:**

Standardized approach to postoperative adjuvant therapy for hepatocellular carcinoma (HCC) remains elusive. This study endeavors to examine the effects of postoperative PD-1 adjuvant therapy on the short-term and long-term prognosis of patients at a heightened risk of post-surgical recurrence.

**Methods:**

The data of HCC patients who underwent hepatectomy at our center from June 2018 to March 2023 were collected from the hospital database. Propensity score matching (PSM) was employed to perform a 1:1 match between the postoperative anti-PD-1 antibody group and the postoperative non-anti-PD-1 antibody group. Kaplan-Meier method was utilized to compare the overall survival (OS) and recurrence-free survival (RFS) between the two groups. Cox regression analysis was conducted to identify the prognostic factors affecting patient outcomes. Subgroup analyses were performed for different high-risk factors.

**Results:**

Among the 446 patients included in the study, 122 patients received adjuvant therapy with postoperative anti-PD-1 antibodies. After PSM, the PD-1 group had postoperative 1-year, 2-year, 3-year, and 4-year OS rates of 93.1%, 86.8%, 78.2%, and 51.1%, respectively, while the non-PD-1 group had rates of 85.3%, 70.2%, 47.7%, and 30.0%. The PD-1 group had postoperative 1-year, 2-year, 3-year, and 4-year RFS rates of 81.7%, 77.0%, 52.3%, and 23.1%, respectively, whereas the non-PD-1 group had rates of 68.4%, 47.7%, and 25.8% in 1-year, 2-year, 3-year. A multifactorial Cox regression analysis revealed that postoperative PD-1 use was a prognostic protective factor associated with OS and RFS. Subgroup analysis results indicated that HCC patients with high recurrence risks significantly benefited from postoperative anti-PD-1 antibody treatment in terms of OS and RFS.

**Conclusion:**

For HCC patients with high-risk recurrence factors and undergoing hepatectomy, postoperative adjuvant therapy with anti-PD-1 antibodies can effectively improve their survival prognosis.

## Introduction

Hepatocellular carcinoma (HCC) accounts for over 80% of primary liver cancers, making it one of the most common cancer types worldwide. The incidence and mortality rates have been increasing year by year (1, 2). For the majority of HCC patients in many Asian countries, the primary treatment option has been hepatic resection (3, 4). However, for most patients undergoing hepatic resection, the five-year recurrence rate is as high as 50-70%, especially for those with high-risk recurrence factors such as microvascular invasion, portal vein tumor thrombosis, large tumor diameter, multiple tumor nodules, and so on (5, 6). Therefore, effective postoperative adjuvant treatments should be offered to HCC patients after hepatic resection to reduce recurrence and improve long-term survival rates.

Currently, immune checkpoint inhibitors (ICIs) have made significant strides in the treatment of unresectable HCC (7–9). Anti-PD-1 antibodies enhance the body’s immune recognition, enabling T cells to reidentify tumor cells within the body (10). Moreover, responses to ICIs can be sustained over a long duration. For certain other tumors, such as melanoma, esophageal cancer, and gastric cancer, anti-PD-1 antibodies have proven effective in extending patients’ overall survival (OS) and recurrence-free survival (RFS). Hence, the mechanisms of ICIs make them a promising approach for postoperative adjuvant therapy in HCC patients. For patients at high risk of postoperative recurrence, several phase III clinical trials are currently underway (11–13). These trials include postoperative combined therapy with anti-PD-1 antibodies and bevacizumab as adjuvant treatment (NCT03847428 and NCT04102098). Some preliminary results indicate that postoperative PD-1 antibodies can effectively extend the survival of high-risk patients. Researchers like Chen et al (14). believe that HCC patients with portal vein tumor thrombosis (PVTT) or tumors larger than or equal to 5 cm significantly benefit from anti-PD-1 antibody treatment in terms of and OS. After curative resection, HCC may leave behind small disseminated lesions, which often lead to postoperative recurrence and worsened prognosis. However, anti-PD-1 antibodies can modulate the immune environment and increase the number of T cells, eliminating these small lesions (15). Therefore, adjuvant therapy based on anti-PD-1 antibodies holds great promise in preventing recurrence and extending survival.

In this study, our research team aims to investigate whether the use of anti-PD-1 antibodies in postoperative HCC patients can improve prognosis, especially in patients with high-risk recurrence factors. This endeavor seeks to provide additional evidence regarding postoperative adjuvant therapy for HCC patients.

## Materials and methods

### Patient selection

This retrospective study collected data from all patients who underwent curative hepatic resection at Zhongshan People Hospital from June 2018 to June 2023. The study adhered to the requirements of the Helsinki Declaration and received approval from the hospital’s ethics committee [*ZS-20210807]*.

Strict inclusion and exclusion criteria were applied in this study. Inclusion criteria were as follows: (1) age greater than 18, (2) postoperative pathological diagnosis of HCC, (3) R0 resection, (4) tumor detected as the initial occurrence, (5) Child-Pugh class A or B, and (6) ECOG performance status 0-1. Exclusion criteria were as follows: (1) prior history of anticancer treatment, (2) incomplete follow-up records, and (3) non-compliance with drug therapy.

### Definition of high-risk recurrence factors

MVI (Microvascular Invasion) is typically defined as clusters of cancer cells observed within the lumens of vessels lined with endothelial cells under a microscope. It is graded pathologically as M1 and M2. PVTT (Portal Vein Tumor Thrombosis) refers to tumor emboli within the portal vein. In this study, PVTT is primarily categorized into the following types: Type I (vp1): involving the invasion of third-order portal vein branches, Type II (vp2): involving the invasion of second-order portal vein branches, and Type III (vp3): involving the invasion of the main portal vein (16). Satellite nodules are defined as small tumor foci appearing within the liver tissue adjacent to the primary tumor, with a distance of less than 2 centimeters between the tumor foci and the primary tumor. In this study, high-risk recurrence factors are defined as one or more of the following: tumor diameter greater than 5 cm, multifocal tumors, MVI, PVTT, or satellite nodules.

### Preoperative assessment

Preoperatively, a meticulous evaluation and discussion are carried out by the hospital’s Multidisciplinary Team (MDT) to determine the surgical approach. The EGOS (Eastern Cooperative Oncology Group) performance status is used to assess the general condition of patients. Preoperative assessments include abdominal CT and magnetic resonance imaging (MRI). Liver functional reserve is assessed using ICG-15 (Indocyanine Green clearance at 15 minutes). Intraoperatively, intraoperative ultrasound is performed for exploration. All surgeries are conducted by a team of experienced surgeons who have surpassed the learning curve. Major hepatectomy is defined as the resection of three or more liver segments, while minor hepatectomy is defined as the resection of one or two liver segments.

### Adjuvant anti-PD-1 antibody and tyrosine kinase inhibitors treatment

HCC patients with high-risk factors are recommended for adjuvant therapy following liver resection. However, the ultimate decision regarding treatment depends on the patient and their family. Treatment with anti-PD-1 antibodies begins four weeks after surgery at the recommended dosage. Patients receive intravenous anti-PD-1 antibody therapy at 21-day intervals. Patients continue to receive anti-PD-1 antibody treatment until HCC recurrence, the occurrence of severe adverse events, or voluntary withdrawal by the patient. Anti-PD-1 antibodies include camrelizumab, toripalimab, sintilimab, and pembrolizumab. Anti-PD-1 antibodies include camrelizumab, toripalimab, sintilimab, and pembrolizumab. The antibodies currently employed are not approved for postoperative adjuvant use and are considered off-label.

Tyrosine kinase inhibitors (TKIs), including sorafenib, lenvatinib, donafenib, regorafenib, and apatinib, are administered at recommended doses starting four weeks postoperatively. Treatment continues until hepatocellular carcinoma (HCC) recurrence, the onset of severe adverse reactions, or patient-initiated withdrawal. Typically, a treatment cycle spans three weeks, with patients in the Patient-Administered Therapy (PAT) group receiving a minimum of three treatment cycles. During the treatment period, drug interruptions or dose reductions are allowed to minimize drug-related toxicities. Adverse reactions are classified according to the Common Terminology Criteria for Adverse Events (CTCAE) version 5.0 developed by the National Cancer Institute.

### Multiple postoperative follow-up visits

The follow-up team comprises one general surgeon and two specialized follow-up coordinators. All patients who have undergone curative hepatic resection are required to undergo regular follow-up visits to monitor the recurrence of liver cancer, the patient’s survival status, and potential drug-related toxicities. In the first year after surgery, follow-up visits are scheduled every three months, including assessments of liver function and plasma alpha-fetoprotein (AFP) levels. Additionally, relevant imaging studies are conducted. In the second year, the follow-up frequency gradually decreases to semi-annual visits. The diagnosis of recurrence is typically based on the typical radiological manifestations of HCC and changes in relevant tumor markers.

Overall Survival (OS) refers to the time from the date of surgery to the patient’s death or the date of the last follow-up visit. Recurrence-Free Survival (RFS) is defined as the time interval between undergoing curative hepatic resection and the first diagnosis of recurrence or the date of the last follow-up visit. The date of the last follow-up was June 1, 2023.

### Data analysis

Statistical analysis in this study was conducted using R software version 4.0.6, which is available at http://www.R-project.org. Continuous variables following a normal distribution were presented as mean ± standard deviation (SD), while non-normally distributed continuous variables were presented as median and interquartile range (IQR). To compare differences between continuous variables, we employed independent samples t-tests or Mann-Whitney U tests, depending on the specific circumstances. Categorical variables were described using numbers (n) or percentages (%), and comparisons were made using the appropriate chi-squared test or Fisher’s exact test.

To adjust for confounding factors between the two groups, we conducted 1:1 propensity score matching (PSM). Propensity scores are continuous values ranging from 0 to 1 and were generated using binary logistic regression with selected variables. We employed nearest-neighbor matching to match patients in the PD-1 group with those in the non-PD-1 group. Survival curves were constructed using the Kaplan-Meier method, and between-group comparisons were made using the log-rank test. Independent prognostic factors for RFS and OS were determined through univariate and multivariate Cox regression analyses. In the univariate Cox regression, factors with a p-value less than 0.05 were included in the multivariate Cox regression for further analysis.

## Results

### Baseline information of PD-1 and No-PD-1 group before PSM and after PSM

After applying strict inclusion and exclusion criteria, our institution included a total of 446 patients who underwent liver resection surgery in the Department of Hepatobiliary and Pancreatic Surgery from June 2018 to March 2023. Among them, 122 patients received postoperative anti-PD-1 antibody treatment. In the PD-1 group, there were 110 males, accounting for 90.2% of the group. The majority of patients had solitary tumors (74.6%), and 104 patients (85.2%) were infected with the hepatitis B virus. Most patients had good preoperative liver function, with 97 patients (80.2%) classified as Child-Pugh class A. Baseline differences existed in several variables between the two groups, including AST, MVI, PVTT, and CSPH, while the other variables showed no statistical differences. In the group of patients who did not receive anti-PD-1 antibody treatment, 260 patients (80.2%) underwent TKI therapy. In the group receiving anti-PD-1 antibody treatment, 95 patients (77.9%) received TKI therapy.

To balance these baseline differences, a 1:1 PSM was performed on the two groups of patients. This resulted in 122 pairs of patients, with a median follow-up time of 32.6 months. Following PSM, there were no statistical differences in any variables between the two groups. Detailed data distribution can be found in (**Table 1**).

**Table 1:** Baseline characteristics of all HCC patients (before and after PSM).

		Before PSM			After PSM		
Variable		No-PD-1	PD-1	*P*	No-PD-1	PD-1	*P*
N		324	122		122	122	
Age (years)	<60	107 (30.0)	52 (42.6)	0.076	50 (24.6)	52 (42.6)	0.897
	≥60	217 (70.0)	70 (57.4)		72 (75.4)	70 (57.4)	
Sex	Female	40 (12.3)	12 (9.8)	0.512	18 (14.8)	12 (9.8)	0.330
	Male	284 (87.7)	110 (90.2)		104 (85.2)	110 (90.2)	
Tumor number	1	234 (71.2)	91(74.6)	0.635	87 (71.3)	91(74.6)	0.666
	≥2	90 (27.8)	31(25.4)		35 (28.7)	31(25.4)	
Tumor length(cm)	<5	185 (57.1)	61 (50.0)	0.200	58 (47.5)	61 (50.0)	0.798
	≥5	139 (42.9)	61 (50.0)		64 (52.5)	61 (50.0)	
HBV	No	44 (13.6)	18 (14.8)	0.760	20 (16.4)	18 (14.8)	0.860
	Yes	280 (86.4)	104 (85.2)		102 (83.6)	104 (85.2)	
HCV	No	320 (98.8)	119 (97.5)	0.398	118 (97.3)	119 (97.5)	1.000
	Yes	4 (1.2)	3 (2.5)		4 (2.7)	3 (2.5)	
Cirrhosis	No	97 (30.0)	42 (34.4)	0.362	38 (31.1)	42 (34.4)	0.683
	Yes	227 (70.0)	80 (65.6)		84 (68.9)	80 (65.6)	
Child-Pugh	A	285 (88.0)	97 (80.2)	0.046	97 (80.2)	97 (80.2)	1.000
	B	39 (12.0)	24 (19.8)		24 (19.8)	24 (19.8)	
ALT (U/L)	<50	254 (78.4)	90 (73.8)	0.313	96 (88.7)	90 (73.8)	0.452
	≥50	70 (21.6)	32 (26.2)		26 (21.3)	32 (26.2)	
AST (U/L)	<40	189 (58.3)	54 (44.3)	0.010	64 (52.5)	54 (44.3)	0.249
	≥40	135 (41.7)	68 (55.7)		58 (47.5)	68 (55.7)	
GGT (U/L)	<60	136 (42.0)	44 (36.1)	0.280	50 (41.0)	44 (36.1)	0.511
	≥60	188 (58.0)	78 (63.9)		72 (59.0)	78 (63.9)	
ALP (U/L)	<125	215 (66.4)	77 (63.1)	0.577	84 (68.9)	77 (63.1)	0.418
	≥125	109 (33.6)	45 (36.9)		38 (31.1)	45 (36.9)	
Alb (g/L)	<35	118 (36.4)	41 (33.6)	0.658	47 (38.5)	41 (33.6)	0.505
	≥35	206 (63.6)	81 (66.4)		75 (61.5)	81 (66.4)	
AFP (µg/mL)	<400	134 (41.4)	62 (50.8)	0.087	61(50.0)	62 (50.8)	1.000
	≥400	190 (58.6)	60 (49.2)		61 (50.0)	60 (49.2)	
Edmondson-Steiner Grade	I+II	183 (56.5)	56 (45.9)	0.094	58 (47.5)	56 (45.9)	0.898
	III+IV	141 (43.5)	66 (54.1)		64 (52.5)	66 (54.1)	
MVI	No	294 (90.7)	98 (80.3)	0.005	102 (83.6)	98 (80.3)	0.618
	Yes	30 (9.3)	24 (19.7)		20 (16.4)	24 (19.7)	
PVTT	No	266 (82.1)	88 (72.1)	0.025	93 (76.2)	88 (72.1)	0.559
	Yes	58 (17.9)	34 (27.9)		29 (23.8)	34 (27.9)	
CSPH	No	295 (91.0)	98 (80.3)	0.003	96 (78.7)	98 (80.3)	0.874
	Yes	29 (9.0)	24 (19.7)		26 (21.3)	24 (19.7)	
TKI	No	64 (19.8)	27 (22.1)	0.599	21 (17.2)	27 (22.1)	0.421
	Yes	260 (80.2)	95 (77.9)		101 (82.8)	95 (77.9)	
Anti-PD-1 antibody							
	camrelizumab	/	5 (4.1)		/	5 (4.1)	
	toripalimab	/	37 (30.3)		/	37 (30.3)	
	sintilimab	/	46 (37.7)		/	46 (37.7)	
	pembrolizumab	/	34 (27.9)		/	34 (27.9)	

AST, aspartate aminotransferase; ALT, alanine aminotransferase; GGT, gamma glutamyl transpeptidase; ALP, alkaline phosphatase; Alb, albumin; AFP, alpha fetoprotein; HBV, hepatitis B virus; HCV, hepatitis C virus; MVI, Microvascular Infiltration; PVTT, portal vein tumor thrombus; CSPH, clinically significant portal hypertension; TKI, Tyrosine kinase inhibitors.

### Univariate and multivariate COX regression to determine prognostic factors affecting overall survival in HCC patients

The results of the univariate Cox regression analysis showed that Tumor length (HR=1.284, 95% CI: 1.198-1.423), AFP (HR=1.784, 95% CI: 1.366-2.164), Differentiation (HR=1.664, 95% CI: 1.287-2.102), MVI (HR=2.172, 95% CI: 1.618-2.914), PVTT (HR=1.899, 95% CI: 1.573-2.223), and CSPH (HR=1.428, 95% CI: 1.132-1.921) were risk factors affecting patient OS. Postoperative use of anti-PD-1 antibodies (HR=0.448, 95% CI: 0.335-0.598) was a protective factor affecting patient OS. When these variables were included in a multivariate Cox regression model, the results of the multivariate Cox regression analysis still indicated that postoperative use of anti-PD-1 antibodies (HR=0.471, 95% CI: 0.367-0.613) remained a protective factor affecting patient OS (**Table 2**).

**Table 2:** Univariate and multivariate Cox regression analysis was used to identify independent risk factors for OS in overall patients.

Variables	HR comparison	UV HR (95% *CI*)	UV p	MV HR (95% *CI*)	MV p*
Age	≥60 vs < 60 years	1.033(0.697-1.288)	0.467		
Gender	Male vs. female	0.975(0.602-1.233)	0.278		
Tumor number	≥2 vs < 2	1.078(0.989-1.315)	0.051		
Tumor length	>5cm vs ≤5cm	1.284(1.198-1.423)	0.023	1.188(1.077-1.352)	0.011
HBV	Yes vs. no	0.961(0.655-1.482)	0.316		
HCV	Yes vs. no	1.102(0.744-1.682)	0.221		
Cirrhosis	Yes vs. no	1.202(0.906-1.413)	0.874		
Child Pugh	B vs A	1.233(0.513-1.806)	0.811		
ALT	≥50 vs<50 U/L	1.022 (0.974-1.321)	0.133		
AST	≥40 vs<40 U/L	1.044(0.980-1.076)	0.157		
GGT	≥45 vs<45 U/L	1.334(0.851-1.740)	0.168		
ALP	≥40 vs<40 U/L	1.007(0.812-1.079)	0.413		
Alb	≥35 vs<35 g/L	0.998 (0.797-1.352)	0.134		
AFP	≥400 vs<400ng/mL	1.784(1.366-2.164)	<0.001	1.588(1.344-1.921)	<0.001
Differentiation	III+IV vs I+II	1.664(1.287-2.102)	<0.001	1.781(1.413-2.134)	<0.001
MVI	Yes vs No	2.172(1.618-2.914)	<0.001	2.003(1.752-2.433)	<0.001
PVTT	Yes vs No	1.899(1.573-2.223)	<0.001	2.071(1.613-2.530)	<0.001
CSPH	Yes vs No	1.428(1.132-1.921)	<0.001	1.315(1.211-1.528)	<0.001
PD-1	Yes vs No	0.448(0.335-0.598)	<0.001	0.471(0.367-0.613)	<0.001

AST, aspartate aminotransferase; ALT, alanine aminotransferase; GGT, gamma glutamyl transpeptidase; ALP, alkaline phosphatase; Alb, albumin; AFP, alpha fetoprotein; HBV, hepatitis B virus; HCV, hepatitis C virus; MVI, Microvascular Infiltration; PVTT, portal vein tumor thrombus; CSPH, clinically significant portal hypertension.

*Those variables found significant at p < 0.05 in univariable analyses were entered into multivariable Cox-regression analyses.

### Univariate and multivariate COX regression to determine prognostic factors affecting recurrence-free survival in patients

The results of the univariate Cox regression analysis revealed that Tumor length (HR=1.319, 95% CI: 1.214-1.520), AFP (HR=1.578, 95% CI: 1.277-2.088), Differentiation (HR=1.563, 95% CI: 1.195-2.261), MVI (HR=2.061, 95% CI: 1.505-2.771), PVTT (HR=1.971, 95% CI: 1.711-2.199), and CSPH (HR=1.581, 95% CI: 1.261-1.986) were risk factors influencing patient RFS. Postoperative use of anti-PD-1 antibodies (HR=0.443, 95% CI: 0.322-0.598) was identified as a protective factor affecting patient RFS. When these variables were included in a multivariate Cox regression model, the results of the multivariate Cox regression analysis continued to show that postoperative use of anti-PD-1 antibodies (HR=0.503, 95% CI: 0.398-0.715) remained a protective factor influencing patient RFS (**Table 3**).

**Table 3:** Univariate and multivariate Cox regression analysis was used to identify independent risk factors for RFS in overall patients.

Variables	HR comparison	UV HR (95% *CI*)	UV p	MV HR (95% *CI*)	MV p*
Age	≥60 vs < 60 years	1.213(0.812-1.431)	0.277		
Gender	Male vs. female	0.911(0.577-1.167)	0.346		
Tumor number	≥2 vs < 2	1.178(0.890-1.377)	0.087		
Tumor length	>5cm vs ≤5cm	1.319(1.214-1.520)	0.007	1.192(1.069-1.423)	0.017
HBV	Yes vs. no	0.877(0.610-1.108)	0.643		
HCV	Yes vs. no	1.216(0.832-1.710)	0.173		
Cirrhosis	Yes vs. no	1.288(0.816-1.713)	0.682		
Child Pugh	B vs A	1.271(0.877-1.578)	0.731		
ALT	≥50 vs<50 U/L	1.018 (0.964-1.287)	0.108		
AST	≥40 vs<40 U/L	1.055(0.991-1.123)	0.341		
GGT	≥45 vs<45 U/L	1.187(0.798-1.512)	0.138		
ALP	≥40 vs<40 U/L	1.038(0.914-1.288)	0.248		
Alb	≥35 vs<35 g/L	0.996 (0.745-1.400)	0.390		
AFP	≥400 vs<400ng/mL	1.578(1.277-2.088)	<0.001	1.473(1.187-2.003)	<0.001
Differentiation	III+IV vs I+II	1.563(1.195-2.261)	<0.001	1.619(1.583-2.278)	<0.001
MVI	Yes vs No	2.061(1.505-2.771)	<0.001	1.964(1.618-2.355)	<0.001
PVTT	Yes vs No	1.971(1.711-2.199)	<0.001	2.048(1.764-2.490)	<0.001
CSPH	Yes vs No	1.581(1.261-1.986)	<0.001	1.444(1.190-1.820)	<0.001
PD-1	Yes vs No	0.443(0.322-0.609)	<0.001	0.503(0.398-0.715)	<0.001

AST, aspartate aminotransferase; ALT, alanine aminotransferase; GGT, gamma glutamyl transpeptidase; ALP, alkaline phosphatase; Alb, albumin; AFP, alpha fetoprotein; HBV, hepatitis B virus; HCV, hepatitis C virus; MVI, Microvascular Infiltration; PVTT, portal vein tumor thrombus; CSPH, clinically significant portal hypertension.

*Those variables found significant at p < 0.05 in univariable analyses were entered into multivariable Cox-regression analyses.

### Prognosis of HCC patients with postoperative use of anti-PD-1 antibodies versus no anti-PD-1 antibodies

**Figure 1:**
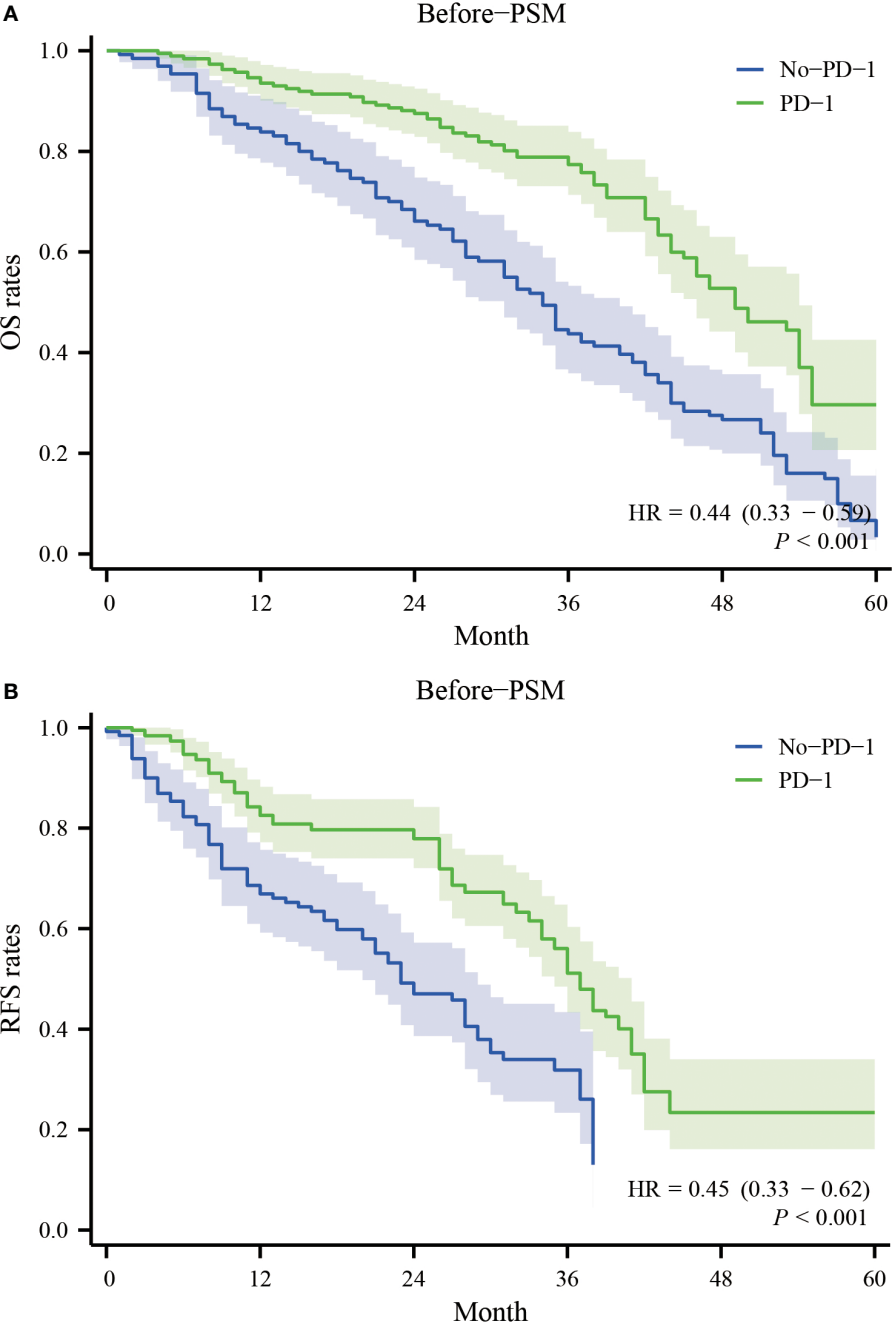
Survival curves for OS and RFS in the PD-1 and No-PD-1 groups before PSM. **(A)** represents the overall survival rate of the two groups of patients before PSM; **(B)** represents the recurrence-free survival rate of the two groups of patients before PSM.

Before PSM, the patients in the PD-1 group had one-year, two-year, three-year, and four-year overall survival rates of 93.5%, 87.5%, 77.4%, and 48.8%, respectively. In contrast, the patients in the No-PD-1 group had one-year, two-year, three-year, and four-year overall survival rates of 84.9%, 69.9%, 43.9%, and 26.6%, respectively. There was a significant statistical difference between the two groups (P<0.001) (**Figure 1A**). Regarding RFS, before PSM, the PD-1 group had one-year, two-year, three-year, and four-year RFS rates of 82.5%, 77.9%, 51.0%, and 22.8%, respectively, while the No-PD-1 group had one-year, two-year, and three-year RFS rates of 69.0%, 48.5%, and 26.4%, respectively. There was a significant statistical difference between the two groups (P<0.001) (**Figure 1B**).

**Figure 2:**
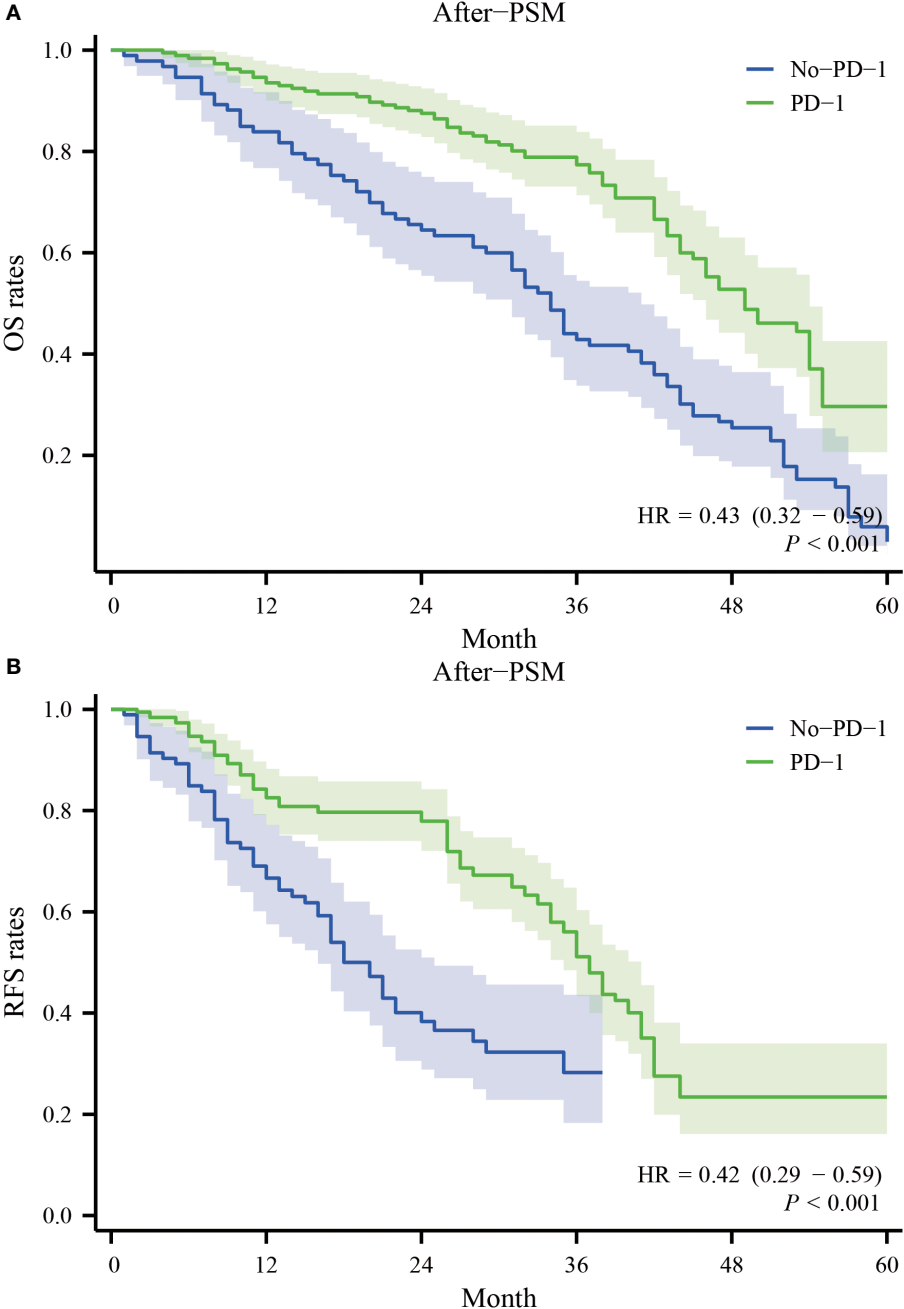
Survival curves for OS and RFS in the PD-1 and No-PD-1 groups after PSM. **(A)** represents the overall survival rate of the two groups of patients after PSM; **(B)** represents the recurrence-free survival rate of the two groups of patients after PSM.

After PSM, the patients in the PD-1 group had one-year, two-year, three-year, and four-year overall survival rates of 93.1%, 86.8%, 78.2%, and 51.1%, respectively. In contrast, the patients in the No-PD-1 group had one-year, two-year, three-year, and four-year overall survival rates of 85.3%, 70.2%, 47.7%, and 30.0%, respectively. There was a significant statistical difference between the two groups (P<0.001) (**Figure 2A**). Regarding RFS, after PSM, the PD-1 group had one-year, two-year, three-year, and four-year RFS rates of 81.7%, 77.0%, 52.3%, and 23.1%, respectively, while the No-PD-1 group had one-year, two-year, and three-year RFS rates of 68.4%, 47.7%, and 25.8%, respectively. There was a significant statistical difference between the two groups (P<0.001) (**Figure 2B**).

An analysis of the causes of death for all deceased patients revealed no statistically significant differences in the proportions of different causes of death between the PD-1 group and the No-PD-1 group. The majority of patient deaths were attributed to the tumor itself, such as postoperative metastasis or recurrence (**Supplementary Table 1**).

### Subgroup analysis based on high-risk factors

**Figure 3:**
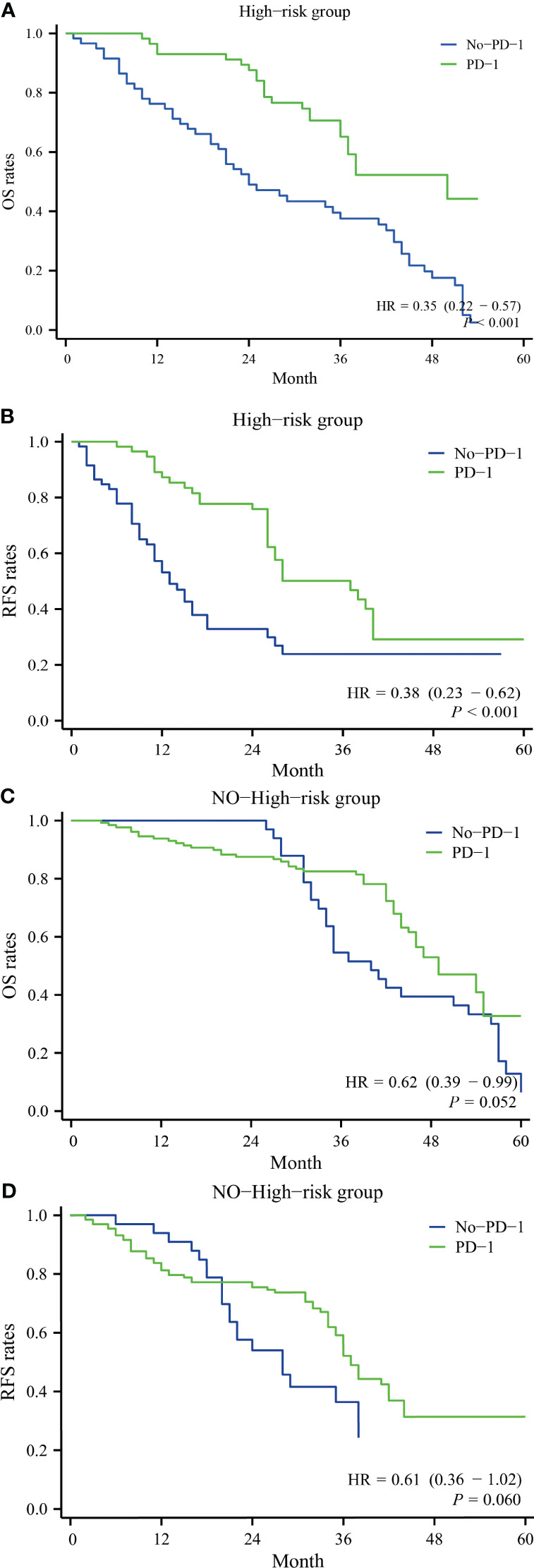
Comparison of survival with and without postoperative anti-PD-1 antibody use in subgroup analyses in the high recurrence risk group or in the non-high recurrence risk group. **(A)** represents the overall survival rate of patients with a high-risk factor in both groups, **(B)** represents the recurrence-free survival rate of patients with a high-risk factor in both groups; **(C)** represents the overall survival rate of patients without a high-risk factor in both groups, and **(D)** represents the recurrence-free survival rate of patients without a high-risk factor in both groups.

Based on the presence or absence of high-risk factors, two groups were formed: the high-risk factor group and the non-high-risk factor group. In the high-risk factor group, for overall survival, the PD-1 group had one-year, two-year, three-year, and four-year overall survival rates of 100.0%, 83.2%, 61.3%, and 52.7%, respectively, while the No-PD-1 group had one-year, two-year, three-year, and four-year overall survival rates of 78.2%, 43.0%, 38.8%, and 18.9%, respectively. There was a significant statistical difference between the two groups (P<0.001) (**Figure 3A**). For recurrence-free survival, in the high-risk factor group, the PD-1 group had one-year, two-year, three-year, and four-year recurrence-free survival rates of 88.1%, 78.6%, 50.3%, and 36.1%, respectively, while the No-PD-1 group had one-year, two-year, three-year, and four-year recurrence-free survival rates of 51.7%, 36.9%, 23.6%, and 23.6%, respectively. Again, there was a significant statistical difference between the two groups (P<0.001) (**Figure 3B**). In the non-high-risk factor group, for overall survival, the PD-1 group had one-year, two-year, three-year, and four-year overall survival rates of 93.2%, 86.9%, 80.8%, and 54.9%, respectively, while the No-PD-1 group had one-year, two-year, three-year, and four-year overall survival rates of 100.0%, 100.0%, 52.2%, and 40.1%, respectively. There was no statistically significant difference between the two groups (P=0.052) (**Figure 3C**). For recurrence-free survival, in the non-high-risk factor group, the PD-1 group had one-year, two-year, three-year, and four-year recurrence-free survival rates of 84.2%, 77.8%, 50.7%, and 35.5%, respectively, while the No-PD-1 group had one-year, two-year, three-year recurrence-free survival rates of 92.3%, 57.7%, and 38.0%, respectively. Similarly, there was no statistically significant difference between the two groups (P=0.060) (**Figure 3D**).

### Adverse effects in patients in the postoperative anti-PD-1 antibody group

All postoperative 122 patients with anti-PD-1 antibodies had a very small number of grade 3-4 AEs, only the postoperative patients with grade 3-4 ALT/AST elevations were relatively higher at 14.8%, the rest had relatively few low-grade hyponatremia, and no patients had Rash, Pruritus, Decreased appetite. Pneumonia, Decreased weight, Nausea/vomiting (**Table 4**). After comparing postoperative complications between the PD-1 group and the No-PD-1 group, it was observed that the proportion of patients with elevated ALT/AST levels was higher in the PD-1 group. Additionally, a higher proportion of patients in the PD-1 group experienced a decrease in neutrophil count. There were no statistically significant differences in other complications (**Supplementary Table 2**).

**Table 4:** AEs in the PD-1 group.

Grade	0, n (%)	1–2, n (%)	3-4, n(%)
Hypoalbuminaemia n (%)	95(77.9)	24(19.7)	3(2.4)
Elevated ALT/AST n (%)	46(37.7)	58(47.5)	18(14.8)
Hyponatremia n (%)	115(94.3)	6(4.9)	1(0.8)
Hypopotassaemia n (%)	110(90.2)	12(9.8)	0(0.0)
Anemia n (%)	79(64.8)	30(24.6)	3(2.6)
Decreased neutrophils n (%)	89(73.0)	28(23.0)	5(4.0)
Decreased white blood cell n (%)	90(73.8)	32(26.2)	0(0.0)
Decreased platelet n (%)	98(80.3)	22(18.0)	2(1.7)
Rash n (%)	122(100.0)	0(0.0)	0(0.0)
Pruritus n (%)	122(100.0)	0(0.0)	0(0.0)
Diarrhea n (%)	121 (99.2)	1(0.8)	0(0.0)
Decreased appetite n (%)	122(100.0)	0(0.0)	0(0.0)
Pneumonia, n (%)	122(100.0)	0(0.0)	0(0.0)
Fatigue n(%)	121 (99.2)	1(0.8)	0(0.0)
Decreased weight n(%)	122(100.0)	0(0.0)	0(0.0)
Nausea/vomiting n(%)	122(100.0)	0(0.0)	0(0.0)

AE, adverse event; ALT, alanine aminotransferase; AST, aspartate aminotransferase.

## Discussion

Currently, there is no standardized approach for adjuvant therapy following curative resection in HCC patients (17). Previous studies have suggested that the efficacy of postoperative adjuvant treatments may be limited. A global multicenter RCT in 2016 demonstrated that sorafenib, for example, did not effectively improve the prognosis of HCC patients after surgery (18). However, the recent performance of anti-PD-1 antibody inhibitors has shown promising results. These inhibitors have the ability to enhance the patient’s immune system and restore its ability to eliminate tumor cells. Given their excellent performance in multiple tumor types, investigating their role in HCC patients after surgery, particularly in those with high-risk recurrence factors, is crucial. The high recurrence rate of HCC remains a significant factor affecting postoperative survival (19, 20). Therefore, it is essential to provide appropriate postoperative adjuvant therapy to improve the survival of high-risk recurrence patients.

In previous studies, anti-PD-1 antibodies have shown promising efficacy in the treatment and downstaging of unresectable HCC patients. Researchers such as Zhu et al (21). demonstrated that a combination therapy of TKI (Tyrosine Kinase Inhibitor) and anti-PD-1 antibodies resulted in 10 out of 63 unresectable HCC patients (15.9%) achieving downstaging and undergoing R0 resection. Simultaneously, Xin et al (22). provided evidence that for unresectable patients, a combination of lenvatinib with PD-1 inhibitors, along with TACE (Transarterial Chemoembolization) triple therapy, could lead to favorable survival outcomes with a lower proportion of severe complications post-treatment, ensuring manageable safety. However, there is currently limited research on adjuvant therapy for HCC post-surgery. In our study, both before and after 1:1 PSM, the prognosis of HCC patients who received postoperative anti-PD-1 antibody treatment was significantly better than those who did not. This conclusion aligns with the findings of Chen and colleagues (14), who discovered that postoperative use of anti-PD-1 antibodies could effectively improve the one-year and two-year overall survival and recurrence-free survival rates of HCC patients at high risk of recurrence. Our study, with a larger sample size, also demonstrated improved three-year overall survival and recurrence-free survival rates in patients who received postoperative anti-PD-1 antibodies. Additionally, Li et al (23). showed that postoperative combination therapy of anti-PD-1 antibodies with TKI could effectively enhance the prognosis of HCC patients with high-risk recurrence factors, and overall, it was found to be a safe treatment approach without severe Grade 4/5 toxicities or adverse events. Therefore, in summary, the postoperative use of anti-PD-1 antibodies in HCC patients can effectively improve their prognosis (24, 25). Furthermore, the results of the multivariable Cox regression analysis suggest that the use of anti-PD-1 antibodies is a protective factor for both OS and RFS in patients.

Subsequently, we stratified the patients into high-risk and low-risk groups, and the results clearly demonstrate that in the high-risk group, anti-PD-1 antibodies can significantly improve both OS (Overall Survival) and RFS (Recurrence-Free Survival) with HRs (Hazard Ratios) of 0.35 and 0.38, respectively. The high-risk group had a notably high postoperative recurrence rate, which may be associated with the potential microscopic dissemination and multicentric development that is often not visible before surgery. The use of immune checkpoint inhibitors (ICI) as adjuvant therapy after surgery can help eliminate these disseminated lesions as much as possible. Additionally, the high-risk group included patients with PVTT (Portal Vein Tumor Thrombosis). In countries like China and other Asian regions, a significant number of cancer patients are diagnosed at an advanced stage, and surgical resection remains a common treatment approach. Therefore, current guidelines in these regions suggest that a relatively aggressive approach, including resection or removal of the tumor thrombus, can be considered during treatment (16). Previous research from Asian centers has also indicated that even in the presence of PVTT, surgical treatment can achieve favorable outcomes. To reflect real-world effectiveness, we included PVTT patients who underwent surgery in our study. It’s worth noting that differences in guidelines between Western and Asian countries may impact the generalizability of the results. Chen and colleagues conducted a separate analysis of the PVTT group and found that the use of ICI resulted in a remarkable HR of 0.15 (14), indicating a highly significant effect. However, in the non-high-risk group, the effect of postoperative ICI was not significant, with p-values around 0.050. This may be related to the relatively smaller sample size. In conclusion, we believe that postoperative anti-PD-1 antibodies hold promise as a therapy for HCC patients at high risk of recurrence following liver resection.

Anti-PD-1 antibody therapy symbolizes the forefront of newer research in HCC. Currently, due to the tumor heterogeneity of HCC, different combination therapies are targeted for different types of HCC. A meta-analysis by Li et al (26). indicates that external beam radiotherapy combined with sorafenib shows improved efficacy in the treatment of unresectable hepatocellular carcinoma (HCC). This combined therapy may guide future selections of sorafenib and local treatment. In a multicenter study conducted by Su et al (27)., the effectiveness of external beam radiotherapy (EBRT) versus transcatheter arterial chemoembolization (TACE) for HCC with a tumor diameter ≥ 5 cm was evaluated. They suggest that EBRT, as the primary local treatment for HCC with a diameter ≥ 5 cm, is more effective than TACE. Li et al (28). propose that the combination of PD-1 inhibitors with TACE has a significant impact on HCC. In summary, the treatment landscape for HCC is continually evolving, with combination therapies becoming a standard approach that can further extend the survival period of patients. At the same time, certain biomarkers can effectively predict patient prognosis, such as ALP, ALBI, and ALR scores, among others. They can further assist researchers in assessing prognosis (29–31). Therefore, there will be a greater focus on immunotherapy combinations and response markers in the future (32–37).

As per our knowledge, this study represents the largest sample size and longest follow-up duration in a single-center study of postoperative adjuvant therapy with anti-PD-1 antibodies. It further substantiates that HCC patients with high-risk recurrence factors after surgery can significantly improve their prognosis through immunotherapeutic interventions. Notably, there was a substantial improvement in OS and RFS at one and two years postoperatively. Furthermore, the adverse events associated with this adjuvant therapy were relatively mild, with very few patients experiencing grade 3-4 Adverse Events (AEs), and there were no deaths attributed to post-treatment AEs. However, it is essential to pay attention to patients who undergo certain combined treatments postoperatively, such as postoperative TACE or postoperative TKI therapy. This subgroup of patients may be more prone to experiencing grade 3-4 AEs. Therefore, close monitoring is required during postoperative adjuvant therapy, particularly in the context of combination treatments (38, 39).

This study has several limitations that should be acknowledged. Firstly, it is an observational single-center study, and the PSM cohort is not equivalent to an RCT cohort. The conclusions of this study need further confirmation through future randomized controlled trials. Additionally, the postoperative use of anti-PD-1 antibodies in this study comes from multiple companies, and there may be differences in treatment efficacy between them. Secondly, differences in patient selection criteria between Western and Eastern countries for those undergoing liver resection may impact the generalizability of our results. Lastly, given the higher proportion of hepatitis B virus infection among Asian HCC patients, postoperative antiviral therapy can influence patient outcomes. Therefore, further studies involving a larger number of patients with longer follow-up periods are needed to validate our findings.

## Conclusion

The use of anti-PD-1 antibodies after hepatocellular carcinoma surgery is an important new and effective intervention that significantly improves short- and long-term survival outcomes in HCC patients with high recurrence factors after hepatic resection, while the therapy is safe and reliable.

## Data availability statement

The original contributions presented in the study are included in the article/**Supplementary Material**. Further inquiries can be directed to the corresponding author.

## Ethics statement

The studies involving humans were approved by the Ethics Committee of Zhongshan People Hospital. The studies were conducted in accordance with the local legislation and institutional requirements. Written informed consent for participation in this study was provided by the participants’ legal guardians/next of kin.

## Author contributions

W-QZ: Data curation, Formal Analysis, Writing – original draft, Writing – review & editing. QZ: Conceptualization, Data curation, Project administration, Writing – original draft. LT: Software, Supervision, Writing – review & editing. Z-FG: Investigation, Methodology, Project administration, Writing – review & editing. FT: Investigation, Methodology, Project administration, Writing – review & editing. H-TT: Data curation, Investigation, Project administration, Writing – original draft. KH: Formal Analysis, Methodology, Project administration, Software, Writing – review & editing. W-QC: Conceptualization, Data curation, Writing – original draft, Writing – review & editing.

## References

[1] Ayuso C Rimola J Vilana R Burrel M Darnell A García-Criado Á. Diagnosis and staging of hepatocellular carcinoma (HCC): current guidelines. Eur J Radiol (2018) 101:72–81. doi: 10.1016/j.ejrad.2018.01.025 29571804

[2] Xing R Gao J Cui Q Wang Q. Strategies to improve the antitumor effect of immunotherapy for hepatocellular carcinoma. Front Immunol (2021) 12:783236. doi: 10.3389/fimmu.2021.783236 34899747 PMC8660685

[3] Allaire M Goumard C Lim C Cleach Le A Wagner M Scatton O. New frontiers in liver resection for hepatocellular carcinoma. JHEP Rep Innovation Hepatol (2020) 2:100134. doi: 10.1016/j.jhepr.2020.100134 PMC7360891 32695968

[4] Gilles H Garbutt T Landrum J. Hepatocellular carcinoma. Crit Care Nurs Clinics North America (2022) 34:289–301. doi: 10.1016/j.cnc.2022.04.004 36049848

[5] Kim E Viatour P. Hepatocellular carcinoma: old friends and new tricks. Exp Mol Med (2020) 52:1898–907. doi: 10.1038/s12276-020-00527-1 PMC8080814 33268834

[6] Nevola R Ruocco R Criscuolo L Villani A Alfano M Beccia D. Predictors of early and late hepatocellular carcinoma recurrence. World J Gastroenterol (2023) 29:1243–60. doi: 10.3748/wjg.v29.i8.1243 PMC10011963 36925456

[7] de Castria TB Khalil DN Harding JJ, EM Abou-Alfa GK. Tremelimumab and durvalumab in the treatment of unresectable, advanced hepatocellular carcinoma. Future Oncol (London England) (2022) 18:3769–82. doi: 10.2217/fon-2022-0652 PMC13238146 36399155

[8] Rimassa L Finn RS Sangro B. Combination immunotherapy for hepatocellular carcinoma. J Hepatol (2023) 79:506–15. doi: 10.1016/j.jhep.2023.03.003 36933770

[9] Sun L Xu X Meng F Liu Q Wang H Li X. Lenvatinib plus transarterial chemoembolization with or without immune checkpoint inhibitors for unresectable hepatocellular carcinoma: A review. Front Oncol (2022) 12:980214. doi: 10.3389/fonc.2022.980214 36249023 PMC9555078

[10] Liu X Lu Y Qin S. Atezolizumab and bevacizumab for hepatocellular carcinoma: mechanism, pharmacokinetics and future treatment strategies. Future Oncol (London England) (2021) 17:2243–56. doi: 10.2217/fon-2020-1290 33663220

[11] Hack SP Spahn J Chen M Cheng AL Kaseb A Kudo M. IMbrave 050: a Phase III trial of atezolizumab plus bevacizumab in high-risk hepatocellular carcinoma after curative resection or ablation. Future Oncol (London England) (2020) 16:975–89. doi: 10.2217/fon-2020-0162 32352320

[12] Qi W Peng W Qi X Qiu Z Wen T Li C. TIDE: adjuvant tislelizumab plus donafenib combined with transarterial chemoembolization for high-risk hepatocellular carcinoma after surgery: protocol for a prospective, single-arm, phase II trial. Front Oncol (2023) 13:1138570. doi: 10.3389/fonc.2023.1138570 37139154 PMC10149831

[13] Zhang W Zhang B Chen XP. Adjuvant treatment strategy after curative resection for hepatocellular carcinoma. Front Med (2021) 15:155–69. doi: 10.1007/s11684-021-0848-3 33754281

[14] Chen W Hu S Liu Z Sun Y Wu J Shen S. Adjuvant anti-PD-1 antibody for hepatocellular carcinoma with high recurrence risks after hepatectomy. Hepatol Int (2023) 17:406–16. doi: 10.1007/s12072-022-10478-6 36645648

[15] Han Y Liu D Li L. PD-1/PD-L1 pathway: current researches in cancer. Am J Cancer Res (2020) 10:727–42. PMC7136921 32266087

[16] Sun J Guo R Bi X Wu M Tang Z Lau WY. Guidelines for diagnosis and treatment of hepatocellular carcinoma with portal vein tumor thrombus in China (2021 edition). Liver Cancer (2022) 11:315–28. doi: 10.1159/000523997 PMC9294940 35978596

[17] Marrero JA Kulik LM Sirlin CB Zhu AX Finn RS Abecassis MM. Diagnosis, staging, and management of hepatocellular carcinoma: 2018 practice guidance by the american association for the study of liver diseases. Hepatol (Baltimore Md) (2018) 68:723–50. doi: 10.1002/hep.29913 29624699

[18] Bruix J Takayama T Mazzaferro V Chau GY Yang J Kudo M. Adjuvant sorafenib for hepatocellular carcinoma after resection or ablation (STORM): a phase 3, randomised, double-blind, placebo-controlled trial. Lancet Oncol (2015) 16:1344–54. doi: 10.1016/S1470-2045(15)00198-9 26361969

[19] Rajendran L Ivanics T Claasen MP Muaddi H Sapisochin G. The management of post-transplantation recurrence of hepatocellular carcinoma. Clin Mol Hepatol (2022) 28:1–16. doi: 10.3350/cmh.2021.0217 34610652 PMC8755475

[20] Tabrizian P Jibara G Shrager B Schwartz M Roayaie S. Recurrence of hepatocellular cancer after resection: patterns, treatments, and prognosis. Ann Surg (2015) 261:947–55. doi: 10.1097/SLA.0000000000000710 25010665

[21] Zhu XD Huang C Shen YH Ji Y Ge NL Sun Qu XD. Downstaging and resection of initially unresectable hepatocellular carcinoma with tyrosine kinase inhibitor and anti-PD-1 antibody combinations. Liver Cancer (2021) 10:320–9. doi: 10.1159/000514313 PMC8339461 34414120

[22] Xin Y Zhang X Liu N Peng G Huang X Cao X. Efficacy and safety of lenvatinib plus PD-1 inhibitor with or without transarterial chemoembolization in unresectable hepatocellular carcinoma. Hepatol Int (2023) 17:753–64. doi: 10.1007/s12072-023-10502-3 37038024

[23] Li J Wang WQ Zhu RH Lv X Wang JL Liang BY. Postoperative adjuvant tyrosine kinase inhibitors combined with anti-PD-1 antibodies improves surgical outcomes for hepatocellular carcinoma with high-risk recurrent factors. Front Immunol (2023) 14:1202039. doi: 10.3389/fimmu.2023.1202039 37359534 PMC10285103

[24] Li ZX Zhang QF Huang JM Huang SJ Liang HB Chen H. Safety and efficacy of postoperative adjuvant therapy with atezolizumab and bevacizumab after radical resection of hepatocellular carcinoma. Clinics Res Hepatol Gastroenterol (2023) 47:102165. doi: 10.1016/j.clinre.2023.102165 37330005

[25] Yang J Jiang S Chen Y Zhang J Deng Y. Adjuvant ICIs plus targeted therapies reduce HCC recurrence after hepatectomy in patients with high risk of recurrence. Curr Oncol (Toronto Ont) (2023) 30:1708–19. doi: 10.3390/curroncol30020132 PMC9955678 36826093

[26] Li H Wu Z Chen J Su K Guo L Xu K. External radiotherapy combined with sorafenib has better efficacy in unresectable hepatocellular carcinoma: a systematic review and meta-analysis. Clin Exp Med (2023) 23:1537–49. doi: 10.1007/s10238-022-00972-4 PMC10460724 36495367

[27] Su K Wang F Li X Chi H Zhang J He K. Effect of external beam radiation therapy versus transcatheter arterial chemoembolization for non-diffuse hepatocellular carcinoma (≥ 5 cm): a multicenter experience over a ten-year period. Front Immunol (2023) 14:1265959. doi: 10.3389/fimmu.2023.1265959 37818373 PMC10560878

[28] Li H Su K Guo L Jiang Y Xu K Gu T. PD-1 inhibitors combined with antiangiogenic therapy with or without transarterial chemoembolization in the treatment of hepatocellular carcinoma: A propensity matching analysis. J Hepatocellular Carcinoma (2023) 10:1257–66. doi: 10.2147/JHC.S415843 PMC10395511 37538403

[29] Li H Guo L Su K Li C Jiang Y Wang P. Construction and validation of TACE therapeutic efficacy by ALR score and nomogram: A large, multicenter study. J Hepatocellular Carcinoma (2023) 10:1009–17. doi: 10.2147/JHC.S414926 PMC10317537 37405321

[30] Su K Huang W Li X Xu K Gu T Liu Y. Evaluation of lactate dehydrogenase and alkaline phosphatase as predictive biomarkers in the prognosis of hepatocellular carcinoma and development of a new nomogram. J hepatocellular carcinoma (2023) 10:69–79. doi: 10.2147/JHC.S398632 36685113 PMC9850255

[31] Su K Shen Q Tong J Gu T Xu K Li H. Construction and validation of a nomogram for HBV-related hepatocellular carcinoma: A large, multicenter study. Ann Hepatol (2023) 28:101109. doi: 10.1016/j.aohep.2023.101109 37100384

[32] Appleman LJ. Multifactorial, biomarker-based predictive models for immunotherapy response enter the arena. J Natl Cancer Institute (2021) 113:7–8. doi: 10.1093/jnci/djaa077 PMC7781442 32516413

[33] Lemaire V Shemesh CS Rotte A. Pharmacology-based ranking of anti-cancer drugs to guide clinical development of cancer immunotherapy combinations. J Exp Clin Cancer Res CR (2021) 40:311. doi: 10.1186/s13046-021-02111-5 34598713 PMC8485537

[34] Looi CK Chung FF Leong CO Wong SF Rosli R Mai CW. Therapeutic challenges and current immunomodulatory strategies in targeting the immunosuppressive pancreatic tumor microenvironment. J Exp Clin Cancer Res CR (2019) 38:162. doi: 10.1186/s13046-019-1153-8 30987642 PMC6463646

[35] Luo XY Wu KM He XX. Advances in drug development for hepatocellular carcinoma: clinical trials and potential therapeutic targets. J Exp Clin Cancer Res CR (2021) 40:172. doi: 10.1186/s13046-021-01968-w 34006331 PMC8130401

[36] Rotte A. Predictive models for response and survival in patients treated with anti-PD-1 monotherapy or with anti-PD-1 and ipilimumab combination: editorial commentary. Ann Trans Med (2023) 11:227. doi: 10.21037/atm-22-6564 PMC10061453 37007583

[37] Yi M Zheng X Niu M Zhu S Ge H Wu K. Combination strategies with PD-1/PD-L1 blockade: current advances and future directions. Mol Cancer (2022) 21:28. doi: 10.1186/s12943-021-01489-2 35062949 PMC8780712

[38] Baxi S Yang A Gennarelli RL Khan N Wang Z Boyce L. Immune-related adverse events for anti-PD-1 and anti-PD-L1 drugs: systematic review and meta-analysis. BMJ (Clinical Res ed) (2018) 360:k793. doi: 10.1136/bmj.k793 PMC5851471 29540345

[39] Wang Y Zhou S Yang F Qi X Wang X Guan X. Treatment-related adverse events of PD-1 and PD-L1 inhibitors in clinical trials: A systematic review and meta-analysis. JAMA Oncol (2019) 5:1008–19. doi: 10.1001/jamaoncol.2019.0393 PMC6487913 31021376

