# Supplementary material for: Postoperative adjuvant immunotherapy for high-risk hepatocellular carcinoma patients
Source: Front Oncol. 2023 Dec 15;13:1289916. doi: 10.3389/fonc.2023.1289916 (PMC10766105; doi:10.3389/fonc.2023.1289916)
Supplement: Supplementary file 1 [file Table_1.docx]

**Supplementary Table 1.** Causes of death

| Deaths from all causes | No-PD-1 (n=259) | PD-1 (n=80) | P |
| --- | --- | --- | --- |
| Tumor causes such as metastasis or recurrence | 207 | 67 | 0.518 |
| Liver failure | 31 | 10 | 0.847 |
| Gastrointestinal hemorrhage | 3 | 1 | 1.000 |
| Rupture of esophageal/gastric varices | 1 | 0 | 1.000 |
| Surgery | 3 | 1 | 1.000 |
| Other | 14 | 1 | 0.209 |
