# Supplementary material for: Postoperative adjuvant immunotherapy for high-risk hepatocellular carcinoma patients
Source: Front Oncol. 2023 Dec 15;13:1289916. doi: 10.3389/fonc.2023.1289916 (PMC10766105; doi:10.3389/fonc.2023.1289916)
Supplement: Supplementary file 2 [file Table_2.docx]

Supplementary Table 2. Postoperative complications in the PD-1 group versus the Non-PD-1 group

| Grade | No-PD-1, n (%) | PD-1 n (%) | P |
| --- | --- | --- | --- |
| Hypoalbuminaemia n (%) | 64(19.8) | 27(22.1) | 0.599 |
| Elevated ALT/AST n (%) | 129(39.8) | 76(62.3) | <0.001 |
| Hyponatremia n (%) | 26(8.0) | 7(5.7) | 0.543 |
| Hypopotassaemia n (%) | 34(10.5) | 12(9.8) | 0.863 |
| Anemia n (%) | 94(29.0) | 33(27.0) | 0.725 |
| Decreased neutrophils n (%) | 35(10.8) | 33(27.0) | <0.001 |
| Decreased white blood cell n (%) | 84(25.9) | 32(26.2) | 0.620 |
| Decreased platelet n (%) | 52(16.0) | 24(19.7) | 0.397 |
| Rash n (%) | 0(0.0) | 0(0.0) | 1.000 |
| Pruritus n (%) | 0(0.0) | 0(0.0) | 1.000 |
| Diarrhea n (%) | 3(0.9) | 1(0.8) | 1.000 |
| Decreased appetite n (%) | 0(0.0) | 0(0.0) | 1.000 |
| Pneumonia, n (%) | 0(0.0) | 0(0.0) | 1.000 |
| Fatigue n(%) | 2(0.6) | 1(0.8) | 1.000 |
| Decreased weight n(%) | 0(0.0) | 0(0.0) | 1.000 |
| Nausea/vomiting n(%) | 0(0.0) | 0(0.0) | 1.000 |

AE: adverse event; ALT: alanine aminotransferase; AST: aspartate aminotransferase
